# Differential Nutrient Inadequacy Among Vietnamese Youth: Results of a Multi-Location and Multi-Group 24-Hour Recall Survey

**DOI:** 10.3390/nu18010130

**Published:** 2025-12-31

**Authors:** Xuan Thi Thanh Le, Huy Duc Do, Quan Thi Pham, Lieu Thi Thu Nguyen, Le Minh Giang, Huong Thi Le

**Affiliations:** 1 School of Preventive Medicine and Public Health, Hanoi Medical University, Hanoi 100000, Vietnam; lethithanhxuan@hmu.edu.vn (X.T.T.L.); phamthiquan@hmu.edu.vn (Q.T.P.); leminhgiang@hmu.edu.vn (L.M.G.); lethihuong@hmu.edu.vn (H.T.L.); 2 College of Health Sciences, VinUniversity, Hanoi 100000, Vietnam; huydd1905@gmail.com; 3 National Hospital of Obstetrics and Gynecology, Hanoi 100000, Vietnam

**Keywords:** dietary intake, micronutrient inadequacy, 24 h recall, youth, Vietnam

## Abstract

**Background**: Vietnam is undergoing a rapid nutrition transition, yet evidence on nutrient intake and inadequacy among adolescents and young adults remains limited. This study aimed to assess nutrient intakes and patterns of inadequacy among Vietnamese youth aged 16–25 years across population groups and regions. **Methods**: A cross-sectional study was conducted among 1005 participants from five provinces in northern, central, and southern Vietnam. Dietary intake was assessed using a two-stage 24 h recall, and nutrient inadequacy was evaluated using Estimated Average Requirement (EAR), Acceptable Macronutrient Distribution Range (AMDR), and Estimated Energy Requirement (EER) reference standards. **Results**: Energy and macronutrient intakes differed across groups. University students had the lowest energy intake, while young workers consumed the highest proportion of carbohydrates. Calcium inadequacy exceeded 95% in all subgroups. Regional disparities were observed, with lower intakes of several micronutrients in the South. Compared with high school students, university students showed higher risks of inadequate protein and vitamin A intake, whereas young workers exhibited lower risks of inadequate carbohydrate and folate intake but a higher risk of vitamin A inadequacy. **Conclusions**: Vietnamese youth exhibited substantial micronutrient inadequacies with marked variation across groups and regions. These findings underscore the need for targeted nutrition interventions tailored to specific youth contexts.

## 1. Introduction

Adolescence and early adulthood are nutritionally important stages characterized by rapid physical growth, higher nutrient demands, and the development of lifelong eating patterns [1,2]. Inadequate nutrition during this critical window can impair linear growth and cognitive development, compromise academic and occupational performance, and increase the long-term risk of noncommunicable diseases [3].

Over the past three decades, Vietnam has undergone one of the most rapid nutrition transitions in Southeast Asia, marked by increased consumption of animal-source foods, edible oils, and ultra-processed products, while persistent micronutrient deficiencies remain widespread [4,5]. Although national surveys have documented substantial reductions in stunting and underweight among young children, anemia, zinc deficiency, and suboptimal vitamin A status continue to affect a significant proportion of adolescents [6]. However, data on nutrient intakes among older adolescents and young adults aged 16–25 years remain limited, as existing surveys primarily focus on children under five [7].

This age group faces distinct challenges that profoundly influence dietary patterns, including greater autonomy in food selection, academic and occupational stress, limited access to cooking facilities (particularly among university students and early workforce entrants), irregular eating schedules, and the widespread adoption of restrictive dieting practices, especially among females [8,9]. Moreover, Vietnam exhibits marked north–south and urban–rural disparities in food availability, culinary traditions, and socioeconomic conditions, which contribute to considerable regional variation in dietary intake [10]. To date, no large-scale study has examined nutrient intakes across both educational/occupational subgroups and geographic regions in Vietnamese youth aged 16–25 years.

Although artificial intelligence-assisted dietary assessment tools such as image-assisted and fully image-based methods have advanced rapidly, their accuracy depends heavily on large annotated local food-image databases and thorough validation against culturally specific mixed dishes [11,12]. These prerequisites remain limited in many low- and middle-income countries, including Vietnam, where diverse cooking practices and complex composite dishes challenge automated portion estimation. In this context, interviewer-administered multiple-pass 24 h dietary recalls supported by a country-specific photographic food atlas continue to offer a robust and culturally appropriate approach for estimating group-level dietary intake.

Therefore, this study aimed to describe differences in daily energy, macronutrient, and selected micronutrient intakes across population groups and regions among Vietnamese youth aged 16–25 years.

## 2. Materials and Methods

### 2.1. Study Design and Participants

A cross-sectional study was conducted from December 2023 to June 2024 in five provinces representing the three major regions of Vietnam: northern (Hanoi, Thai Nguyen), central (Da Nang), and southern (Ho Chi Minh City, Binh Duong). Within each province, study sites (high schools, universities, and factories/workplaces) were purposively selected in collaboration with local authorities such as the Department of Health and Department of Education and Training. Participants aged 16–25 years were then recruited from these selected sites through an on-site, convenience-based approach without random selection of locations or individuals.

The sample size was calculated using the formula for estimating a population proportion with relative precision:

$$n=Z_{1-\alpha /2}^{2}\frac{p(1-p)}{{\left(p\cdot \varepsilon \right)}^{2}}$$

where

- *n*: Minimum required sample size.
- *p*: is the estimated proportion of students who met the recommended protein intake; we used p = 0.45 to calculate the sample size for a single proportion based on previous research [13].
- *ε*: Relative precision, set at 0.12 in this study.
- *z*: Confidence level coefficient; with *α* = 0.05, *z*(1 − *α*/2) = 1.96.

The minimum sample size was first estimated as 327 participants using the formula for a single population proportion. To account for the multistage cluster sampling design, the sample size was multiplied by a design effect of 2. After allowing a 10–15% margin for non-response, the final required sample size was approximately 720–770 participants. In practice, 1005 participants were recruited.

### 2.2. Dietary Assessment

A two-stage 24 h dietary recall protocol was used to enhance accuracy and minimize participant burden. Participants first completed a structured, self-administered online 24 h recall accessed via a secure link. The online tool included an instructional video, photographs of common Vietnamese dishes and regional foods, and images of household utensils and portion sizes to assist estimation.

Within 0.5–4 h of completing the online recall, trained nutritionists conducted a comprehensive face-to-face interviewer-administered 24 h dietary recall using a standardized multiple-pass approach [14]. Portion sizes were estimated with support from the Vietnamese Photographic Atlas of Food Portions developed by the National Institute of Nutrition, along with commonly used household utensils and food models [15]. The prior online recall was used as a memory aid to facilitate probing, identify discrepancies, and clarify ambiguous food items. All interviewers received 5 days of intensive training and standardization to ensure consistency in data collection.

Nutrient intakes were calculated exclusively from the interviewer-administered recall using the Vietnamese Food Composition Table (2017) [16]. Nutrient intakes were derived solely from the interviewer-administered recall, which provides greater accuracy than self-reported online recalls in transitional settings while benefiting from the online pre-fill as a memory prompt. All foods reported during the interviewer-administered 24 h recall were converted to raw edible weights and energy equivalents immediately after each interview.

### 2.3. Anthropometry and Sociodemographic Data

Height and weight were measured onsite at participating schools, universities, and workplaces during the field visit, prior to dietary data collection and on the same day as the interviewer-administered 24 h recall. Body weight was measured to the nearest 0.1 kg using a calibrated digital scale (Sailaza SA-2312, Sailaza Co., Ltd., Da Nang, Vietnam), and standing height was measured to the nearest 0.1 cm using a portable stadiometer (SECA 213, SECA GmbH & Co. KG, Hamburg, Germany). Participants were measured without shoes and heavy clothing. All anthropometric measurements were conducted by trained investigators following standardized measurement protocols [17].

Sociodemographic information, including age, sex, region, place of origin (urban, rural, or mountainous), and educational/occupational status (high school student, university student, or young worker), was collected through direct face-to-face interviews using a structured questionnaire.

The sequence of data collection was as follows: participants first underwent anthropometric measurements (height and weight). They then completed the online self-administered 24 h dietary recall. Within 0.5–4 h after completing the online recall, trained interviewers conducted a face-to-face 24 h dietary recall to verify, probe, and clarify dietary information.

### 2.4. Reference Standards and Definitions of Inadequacy

Individual Estimated Energy Requirement (EER) was computed using the Institute of Medicine equations for active physical activity level [18]. Macronutrient balance was evaluated against age-specific Acceptable Macronutrient Distribution Ranges (AMDR) [19]. Micronutrient inadequacy was defined as intake below the age- and sex-specific Estimated Average Requirement (EAR), based on the Dietary Reference Intakes [20]. For nutrients with established EAR values (including calcium), the EAR cut-point method was applied to classify inadequacy.

### 2.5. Statistical Analysis

Dietary intake data were cleaned and converted from cooked food portions to raw edible weights using the conversion tables of the National Institute of Nutrition. Nutrient values of the diets were entered into Microsoft Excel 365 and calculated based on the Vietnamese Food Composition Table (2016) [16]. Data were analyzed using Stata 17.0 (StataCorp, College Station, TX, USA). Nutrient intakes exhibited skewed distributions; medians and interquartile ranges (IQR) are reported. Group differences were tested using Kruskal–Wallis tests for continuous variables and χ^2^ tests for proportions. Multivariable logistic regression was used to estimate adjusted odds ratios (aOR) and 95% confidence intervals for nutrient inadequacy/excess, adjusting for sex, region, and place of origin (urban/rural/mountainous). Two-sided *p*-values <0.05 were considered statistically significant.

### 2.6. Ethical Consideration

The study was approved by the Institutional Review Board of Hanoi Medical University under approval number 991/GCN-HMUIRB dated 30 October 2023. This study was conducted as a component of the national research project ID 08/22-ĐTĐL.XH-XNT. All participants provided written informed consent prior to data collection.

## 3. Results

The distribution of participants differed across the three groups. High school students, university students, and young workers accounted for 32.3%, 49.3%, and 18.4% of the sample, respectively. The median age was 19. The proportion of females was higher among university students, while the sex distribution was more balanced in the other two groups. Participants from the North constituted the largest share of the sample, followed by those from the South and Central regions. Most participants originated from urban areas, with smaller proportions from rural and mountainous areas (Table 1).

Significant differences in energy and macronutrient intake were observed across youth groups (*p* < 0.001). High school students had the highest median energy intake, while university students reported the lowest. Young workers consumed a substantially higher proportion of carbohydrates and the lowest proportion of fat. For micronutrients, vitamin A intake differed markedly, with high school students showing the highest levels (*p* < 0.001), and folate intake was significantly greater among young workers (*p* = 0.01). Calcium, iron, zinc, and vitamin C intakes were similar across groups (Table 2).

Significant regional differences were observed in several nutrient intakes (*p* < 0.001). Participants in the North and Central regions reported higher median energy intake than those in the South. Fat intake was highest in the North, whereas the Central region showed the lowest fat contribution and the highest carbohydrate proportion. For micronutrients, the Central region had the highest median calcium and vitamin A intakes, while the South consistently showed the lowest intakes of calcium, zinc, and folate. Vitamin C intake was highest in the North and lowest in the Central region. Iron intake did not differ significantly across regions (Table 3).

Across youth groups, the prevalence of nutrient inadequacy showed clear variation. University students had the highest proportion of insufficient protein intake, while young workers had the lowest carbohydrate inadequacy. Lipid inadequacy was common in all groups. Calcium inadequacy exceeded 95% in every group. Vitamin A and folate inadequacy were also frequent, with vitamin A inadequacy highest among university students and young workers (Table 4).

Across regions, inadequate intake of several nutrients varied. The South had higher prevalences of inadequate protein, zinc, folate, and vitamin C intake, whereas lipid inadequacy was most common in the Central region. Calcium inadequacy remained consistently high across all regions.

**Figure 1:**
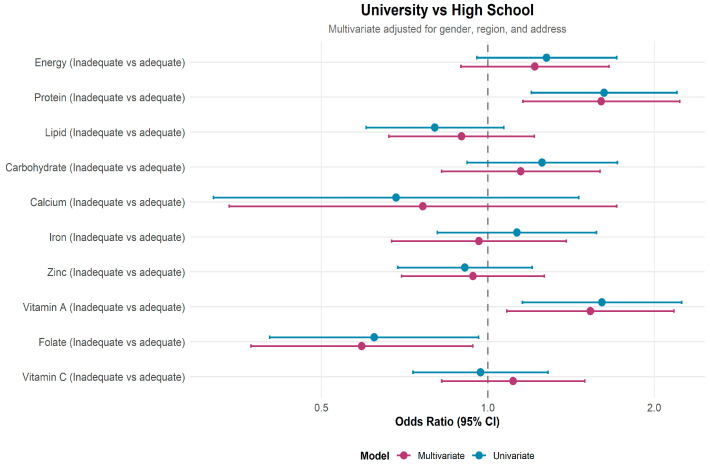
Crude and adjusted odds ratios for nutrient inadequacy among university students compared with high school students, adjusted for gender, region, and place of origin.

Figure 1 presents the crude and adjusted odds ratios for nutrient inadequacy among university students compared with high school students (reference group). After adjustment, university students had higher odds of inadequate protein intake (aOR = 1.61; 95% CI: 1.16–2.23) and vitamin A intake (aOR = 1.53; 95% CI: 1.08–2.17). In contrast, the odds of folate inadequacy were lower among university students (aOR = 0.59; 95% CI: 0.37–0.94). No significant differences were observed between the two groups for energy, lipid, carbohydrate, calcium, iron, zinc, or vitamin C inadequacy.

**Figure 2:**
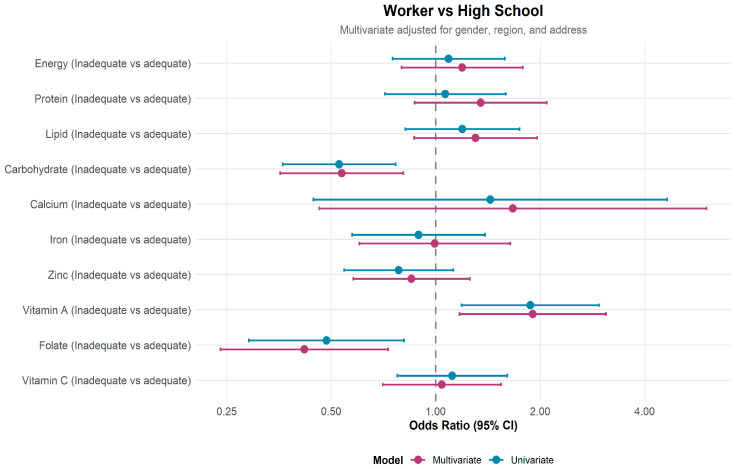
Crude and adjusted odds ratios for nutrient inadequacy among young workers compared with high school students. Adjusted models included gender, region, and place of origin. Young women showed significantly higher inadequacy of protein, carbohydrate, iron, zinc, and folate than males (*p* < 0.05). Calcium and vitamin A inadequacy were prevalent in both sexes (Supplementary Table S1).

Figure 2 presents the crude and adjusted odds ratios for nutrient inadequacy among young workers compared to high school students (reference group). After adjustment for sex, region, and place of origin, young workers had lower odds of inadequate carbohydrate intake (aOR = 0.54; 95% CI: 0.36–0.81) and folate intake (aOR = 0.42; 95% CI: 0.24–0.73). In contrast, the odds of vitamin A inadequacy were higher among young workers (aOR = 1.90; 95% CI: 1.17–3.10). No significant differences were observed for energy, protein, lipid, calcium, iron, zinc, or vitamin C.

## 4. Discussion

This multi-region, multi-group study provides a detailed characterization of daily nutrient intakes among Vietnamese youth aged 16–25 years in the surveyed provinces. The findings indicate consistently low micronutrient intakes across groups, with calcium and folate inadequacy being particularly widespread. Young women exhibited higher prevalences of inadequate intake for most micronutrients, with iron and folate showing the most pronounced differences. This pattern may reflect higher physiological requirements as well as differences in dietary practices, suggesting increased nutritional vulnerability among young women. Additionally, nutritional risk profiles differed across educational and occupational groups, reflecting how lifestyle circumstances during late adolescence and early adulthood may influence dietary patterns. These findings are consistent with reports from other Asian settings undergoing rapid nutrition transitions [21]. However, by examining differences across youth subgroups and country regions, this study provides further insights about the need for targeted nutrition strategies that have not received adequate attention in other studies. This approach highlights within-country variation in nutrient inadequacy that is often obscured in age-aggregated or nationally averaged analyses. Furthermore, the observed patterns of micronutrient inadequacy, particularly the widespread calcium inadequacy across both sexes and the higher prevalence of iron and folate inadequacy among young women, are likely relevant to other low- and middle-income countries undergoing rapid nutrition transitions.

### 4.1. High Prevalences of Calcium and Folate Inadequacy Indicate a Persistent Dietary Gap

Calcium inadequacy affected over 95% of participants, one of the highest rates reported in the region. This aligns with national Vietnamese data showing extremely low dairy consumption and insufficient intake of calcium-rich foods among school-aged children and young women [22,23]. Similar patterns are seen in Asian countries, where traditional diets are naturally low in dairy, and bone-in fish consumption has declined due to urban dietary changes [21].

Folate inadequacy (85.7%) in this sample also parallels biochemical studies documenting folate deficiency among Vietnamese women of reproductive age [24]. Since many individuals in this age group approach their reproductive years, chronic low folate intake is concerning due to its association with neural tube defects and poor pregnancy outcomes [25].

Together, these findings suggest a structural dietary gap in Vietnam’s food system, highlighting an urgent need for population-wide interventions through school-based or workplace-based interventions for youths, such as mandatory folic acid fortification and promotion of affordable calcium-rich foods.

### 4.2. Nutrient Intake Patterns and Inadequacy Risks in University Students

After adjustment for sociodemographic characteristics, university students exhibited significantly higher risks of inadequate protein and vitamin A intake compared with high school students. These findings mirror previous research in Asia and globally, which consistently shows that university students face multiple dietary challenges due to abrupt independence, academic pressure, limited cooking facilities, and financial constraints [26,27].

Convenience-driven consumption of instant noodles, fried snacks, and inexpensive street foods, often poor in high-quality protein and vitamin A, may explain these deficits. Similar trends have been reported in some countries, where students demonstrate lower intake of fruits, vegetables, and animal source foods compared with their peers living at home [28].

The lower prevalence of folate inadequacy among university students may reflect differences in food consumption patterns across age groups; however, this cannot be confirmed without more detailed analyses of dietary sources and frequency of intake. Further research is needed to identify the specific food sources contributing to this pattern among university students in Vietnam.

### 4.3. Influence of Occupational Context on Nutrient Intake in Young Workers

After adjustment for sociodemographic characteristics, young workers had lower odds of inadequate carbohydrate and folate intake compared with high school students. These differences may reflect variation in food access, meal timing, and the broader work environment; however, the specific pathways cannot be established from the present data. Evidence from workplace nutrition research suggests that organizational factors, including scheduled meal breaks, structured eating environments, and the availability of staff canteens, may influence dietary behavior among employees [29]. For example, a recent study in Finland reported that employees who regularly consumed lunch at a staff canteen had higher frequencies of fresh and cooked vegetable intake than those who did not [30]. While this study did not assess meal provision systems or workplace food environments in Vietnam, such contextual factors may help explain the observed nutrient patterns and merit further investigation.

Young workers exhibited higher odds of vitamin A inadequacy, suggesting potential gaps in access to or consumption of vitamin A-rich foods. As the 24 h recall does not capture environmental determinants of food choice, additional qualitative or environmental assessments are needed to understand these differences. No significant disparities were found for other nutrients, indicating that nutrient inadequacy in this group is selective rather than generalized.

Taken together, these findings highlight the importance of considering workplace settings when examining dietary behaviors in young adults. Future research should incorporate measures of workplace meal provision, work schedules, and food environment characteristics to better characterize how occupational contexts shape nutrient intake among young workers.

### 4.4. Regional Disparities and Implications for the Nutrition Transition

Significant regional variation was observed, particularly for zinc, folate, vitamin A, and vitamin C. Participants in the South exhibited substantially higher inadequacy of zinc and folate, whereas those in the Central region had the highest vitamin C inadequacy. These regional differences reflect Vietnam’s uneven nutrition transition: highly urbanized southern areas have greater availability of processed foods, reduced vegetable intake, and higher consumption of ready-to-eat meals [21,31,32].

Similar regional dietary disparities have been documented in Asian countries where rapid urbanization has led to lower micronutrient density despite stable or rising energy intake [33,34]. Our findings underscore the need for geographically tailored policies that account for local food environments and cultural dietary practices.

### 4.5. Strengths and Limitations

This study has several notable strengths. It includes a large and diverse sample of adolescents and young adults across multiple regions of Vietnam, enabling meaningful comparisons between educational/occupational groups and geographic areas. The two-stage dietary assessment approach combining an online pre-filled recall with an interviewer-administered 24 h recall likely improved data completeness and accuracy relative to single-mode recall methods. Standardized nutrient calculations based on the Vietnamese Food Composition Table and the use of internationally recognized reference standards (EAR, AMDR, EER) further enhance the comparability and relevance of the findings.

However, several limitations should be acknowledged. First, due to the cross-sectional design, causal inference is not possible, and all observed associations should be interpreted as correlational. Second, dietary intake was measured using a single 24 h recall per participant, which may not fully capture usual intake and is subject to recall bias and day-to-day variation. Third, the study did not collect data on contextual determinants of diet, such as food availability at home, school, or workplace, canteen meal characteristics, or household food security, limiting the ability to explain observed group and regional differences. Fourth, although the sample is large and diverse, it is not nationally representative; therefore, generalization to all Vietnamese youth should be made with caution. Finally, residual confounding may persist despite adjustment for key sociodemographic factors, as unmeasured variables such as physical activity or socioeconomic status were not fully captured.

## 5. Conclusions

This study identified substantial micronutrient inadequacies among adolescents and young adults in the surveyed provinces, with calcium and folate inadequacy especially widespread. Nutrient risk patterns differed by educational/occupational groups and across regions, reflecting variations in dietary behaviors and local food environments.

Targeted nutrition efforts within schools, universities, and workplaces may help address these gaps by improving access to nutrient-dense foods and supporting healthier eating practices among young people. Future studies using multiple dietary assessment days and incorporating environmental and socioeconomic factors are needed to clarify the determinants of these patterns and to guide more context-specific interventions.

## Figures and Tables

**Table 1:** Sociodemographic characteristics of the study population (n = 1005).

Columns by: Group	High School Students	University Students	Young Workers	Total	*p*-Value *
*n* (%)	325 (32.3)	495 (49.3)	185 (18.4)	1005 (100.0)	
Age, median (IQR)	18.00 (17.00; 18.00) ^a^	20.00 (19.00; 21.00) ^b^	23.00 (21.00; 24.00) ^c^	19.00 (18.00; 21.00)	<0.05
Sex, *n* (%)					
Male	166 (51.1)	190 (38.4)	94 (50.8)	450 (44.8)	<0.05
Female	159 (48.9)	305 (61.6)	91 (49.2)	555 (55.2)	
Region, *n* (%)					
North	141 (43.4)	326 (65.9)	78 (42.2)	545 (54.2)	<0.05
Central	47 (14.5)	62 (12.5)	64 (34.6)	173 (17.2)	
South	137 (42.2)	107 (21.6)	43 (23.2)	287 (28.6)	
Place of origin, *n* (%)					
Urban	235 (72.3)	383 (77.4)	87 (47.0)	705 (70.1)	<0.05
Rural	78 (24.0)	92 (18.6)	86 (46.5)	256 (25.5)	
Mountainous	12 (3.7)	20 (4.0)	12 (6.5)	44 (4.4)	

* Notes: Comparisons among groups were performed using the Kruskal–Wallis test. Statistical significance was defined as *p* < 0.05. ^a, b, c^: Pair wise comparison, values sharing the same superscript letter are not significantly different, whereas values with different superscript letters are significantly different (adjusted *p* < 0.05).

**Table 2:** Daily energy, macronutrient distribution, and selected micronutrient intakes by occupational group among Vietnamese youth aged 16–25 years (n = 1005).

Nutrient	High School Students	University Students	Young Workers	Total	*p*-Value *
*n* (%)	325 (32.3)	495 (49.3)	185 (18.4)	1005 (100.0)	
Energy (E) (kcal/d), median (IQR)	1663.42 (1266.98; 2232.02) ^a^	1516.95 (1093.20; 2031.05) ^b^	1622.30 (1184.25; 2164.35) ^ab^	1579.03 (1174.73; 2124.47)	<0.05
Protein (% of E), median (IQR)	18 (15; 23)	18 (14; 21)	17 (15; 22)	18 (15; 22)	>0.05
Fat (% of E), median (IQR)	30 (22; 37) ^a^	29 (20; 38) ^a^	21 (15; 29) ^b^	28 (19; 36)	<0.05
Carbohydrate (% of E), median (IQR)	50 (42; 60) ^a^	52 (42; 62) ^a^	60 (52; 67) ^b^	54 (43; 63)	<0.05
Calcium (mg/d), median (IQR)	322.29 (198.40; 458.63)	303.71 (175.46; 472.93)	314.90 (217.21; 462.50)	314.90 (193.78; 468.60)	>0.05
Iron (mg/d), median (IQR)	11.69 (8.20; 17.35)	12.22 (7.47; 20.85)	11.50 (7.64; 15.89)	11.90 (7.80; 17.90)	>0.05
Zinc (mg/d), median (IQR)	7.81 (5.50; 11.13)	7.80 (4.58; 11.54)	8.40 (6.10; 11.46)	7.90 (5.19; 11.30)	>0.05
Vitamin A (µg RE/d), median (IQR)	276.72 (26.34; 615.81) ^a^	187.50 (7.20; 422.40) ^b^	160.00 (15.75; 429.15) ^b^	187.50 (11.00; 474.20)	<0.05
Folate (µg/d), median (IQR)	130.90 (63.93; 215.55) ^a^	112.77 (51.92; 237.85) ^a^	150.90 (79.25; 275.98) ^b^	125.78 (60.43; 230.74)	<0.05
Vitamin C (mg/d), median (IQR)	53.90 (24.50; 105.72)	52.37 (22.30; 123.33)	55.30 (28.70; 100.90)	53.90 (24.34; 111.80)	>0.05

* Notes: Comparisons among groups were performed using the Kruskal–Wallis test. Statistical significance was defined as *p* < 0.05. ^a, b^: Pair wise comparison, values sharing the same superscript letter are not significantly different, whereas values with different superscript letters are significantly different (adjusted *p* < 0.05).

**Table 3:** Daily energy, macronutrient distribution and selected micronutrient intakes by region (n = 1005).

Variable	North	Central	South	Total	* *p*-Value
*n* (%)	545 (54.2)	173 (17.2)	287 (28.6)	1005 (100.0)	
Energy (E) (kcal/d), median (IQR)	1634.01 (1209.15; 2209.97) ^a^	1663.42 (1240.91; 2204.07) ^a^	1445.18 (1080.19; 1918.95) ^b^	1579.03 (1174.73; 2124.47)	<0.05
Protein (% of E), median (IQR)	18 (15; 21)	19 (15; 22)	18 (15; 23)	18 (15; 22)	>0.05
Fat (% of E), median (IQR)	29 (21; 38) ^a^	23 (16; 34) ^b^	27 (19; 35) ^c^	28 (19; 36)	<0.05
Carbohydrate (% of E), median (IQR)	52 (42; 61) ^a^	56 (46; 66) ^b^	55 (44; 63) ^b^	54 (43; 63)	<0.05
Calcium (mg/d), median (IQR)	316.68 (190.38; 474.81) ^a^	365.81 (237.80; 554.24) ^b^	285.61 (179.00; 407.00) ^a^	314.90 (193.78; 468.60)	<0.05
Iron (mg/d), median (IQR)	12.63 (7.69; 18.73)	11.82 (8.46; 16.53)	10.90 (7.45; 17.14)	11.90 (7.80; 17.90)	>0.05
Zinc (mg/d), median (IQR)	8.60 (5.50; 12.00) ^a^	8.03 (5.68; 11.65) ^a^	6.93 (4.40; 10.31) ^b^	7.90 (5.19; 11.30)	<0.05
Vitamin A (µg RE/d), median (IQR)	187.50 (8.50; 442.00) ^a^	274.00 (32.59; 534.00) ^b^	188.02 (11.00; 499.73) ^ab^	187.50 (11.00; 474.20)	<0.05
Folate (µg/d), median (IQR)	134.30 (70.90; 249.15) ^a^	134.70 (64.55; 222.95) ^a^	102.86 (47.19; 197.30) ^b^	125.78 (60.43; 230.74)	<0.05
Vitamin C (mg/d), median (IQR)	64.10 (28.23; 124.42) ^a^	42.09 (21.25; 92.58) ^b^	47.17 (21.00; 94.69) ^b^	53.90 (24.34; 111.80)	<0.05

* Notes: Comparisons among regions were performed using the Kruskal–Wallis test. Statistical significance was defined as *p* < 0.05. ^a, b, c^: Pair wise comparison, values sharing the same superscript letter are not significantly different, whereas values with different superscript letters are significantly different (adjusted *p* < 0.05).

**Table 4:** Prevalence of inadequate or excessive macronutrient and micronutrient intake by occupational group and by region among Vietnamese youth aged 16–25 years (n = 1005).

Columns by: Group	Total	High School Students	University Students	Young Workers	* *p*-Value Group	North	Central	South	* *p*-Value Region
n (%)	1005 (100.0)	325 (32.3)	495 (49.3)	185 (18.4)		545 (54.2)	173 (17.2)	287 (28.6)	
Protein, n (%)									
Insufficient	330 (32.8)	89 (27.4)	188 (38.0)	53 (28.6)	<0.05	172 (31.6)	40 (23.1)	118 (41.1)	<0.05
Adequate	654 (65.1)	223 (68.6)	299 (60.4)	132 (71.4)		359 (65.9)	130 (75.1)	165 (57.5)	
Excess	21 (2.1)	13 (4.0)	8 (1.6)	0 (0.0)		14 (2.6)	3 (1.7)	4 (1.4)	
Lipid, n (%)									
Insufficient	609 (60.6)	203 (62.5)	283 (57.2)	123 (66.5)	<0.05	294 (53.9)	116 (67.1)	199 (69.3)	<0.05
Adequate	258 (25.7)	72 (22.2)	137 (27.7)	49 (26.5)		159 (29.2)	38 (22.0)	61 (21.3)	
Excess	138 (13.7)	50 (15.4)	75 (15.2)	13 (7.0)		92 (16.9)	19 (11.0)	27 (9.4)	
Carbohydrate, n (%)									
Insufficient	703 (70.0)	229 (70.5)	371 (74.9)	103 (55.7)	<0.05	385 (70.6)	108 (62.4)	210 (73.2)	>0.05
Adequate	206 (20.5)	68 (20.9)	84 (17.0)	54 (29.2)		106 (19.4)	41 (23.7)	59 (20.6)	
Excess	96 (9.6)	28 (8.6)	40 (8.1)	28 (15.1)		54 (9.9)	24 (13.9)	18 (6.3)	
Calcium adequacy, n (%)									
Inadequate	969 (96.4)	315 (96.9)	473 (95.6)	181 (97.8)	>0.05	526 (96.5)	165 (95.4)	278 (96.9)	>0.05
Adequate	36 (3.6)	10 (3.1)	22 (4.4)	4 (2.2)		19 (3.5)	8 (4.6)	9 (3.1)	
Iron adequacy, n (%)									
Inadequate	233 (23.2)	73 (22.5)	122 (24.6)	38 (20.5)	>0.05	123 (22.6)	32 (18.5)	78 (27.2)	>0.05
Adequate	772 (76.8)	252 (77.5)	373 (75.4)	147 (79.5)		422 (77.4)	141(81.5)	209 (72.8)	
Zinc adequacy, n (%)									
Inadequate	484 (48.2)	165 (50.8)	238 (48.1)	81 (43.8)	>0.05	242 (44.4)	76 (43.9)	166 (57.8)	<0.05
Adequate	521 (51.8)	160 (49.2)	257 (51.9)	104 (56.2)		303 (55.6)	97 (56.1)	121 (42.2)	
Vitamin A adequacy, n (%)									
Inadequate	791 (78.7)	236 (72.6)	401 (81.0)	154 (83.2)	<0.05	435 (79.8)	131 (75.7)	225 (78.4)	>0.05
Adequate	214 (21.3)	89 (27.4)	94 (19.0)	31 (16.8)		110 (20.2)	42 (24.3)	62 (21.6)	
Folate adequacy, n (%)									
Inadequate	861 (85.7)	292 (89.8)	419 (84.6)	150 (81.1)	<0.05	455 (83.5)	147 (85.0)	259 (90.2)	<0.05
Adequate	144 (14.3)	33 (10.2)	76 (15.4)	35 (18.9)		90 (16.5)	26 (15.0)	28 (9.8)	
Vitamin C adequacy, n (%)									
Inadequate	561 (55.8)	181 (55.7)	272 (54.9)	108 (58.4)	>0.05	273 (50.1)	112(64.7)	176 (61.3)	<0.05
Adequate	444 (44.2)	144 (44.3)	223 (45.1)	77 (41.6)		272 (49.9)	61 (35.3)	111 (38.7)	

* Notes Comparisons across occupational groups and across regions were conducted using the Chi-square test. Statistical significance was defined as *p* < 0.05.

## Data Availability

The data presented in this study are available on request from the corresponding author. The data are not publicly available due to ethical and privacy restrictions. Aggregate data may be provided upon reasonable request to the corresponding author, subject to institutional and ethical guidelines.

## Supplementary Materials

The following supporting information can be downloaded at https://www.mdpi.com/article/10.3390/nu18010130/s1. Table S1. Prevalence of inadequate intake of macronutrients and selected micronutrients by sex among Vietnamese youth aged 16–25 years (n = 1005).
