# Supplementary material for: Differential Nutrient Inadequacy Among Vietnamese Youth: Results of a Multi-Location and Multi-Group 24-Hour Recall Survey
Source: Nutrients. 2025 Dec 31;18(1):130. doi: 10.3390/nu18010130 (PMC12788094; doi:10.3390/nu18010130)
Supplement: Supplementary file 1 [file nutrients-18-00130-s001.zip › nutrients-4060960-supplementary.pdf]

Supplementary Table S1. Prevalence of inadequate intake of macronutrients and selected micronutrients by sex among Vietnamese youth aged 16–25 years (n = 1005)

Nutrient adequacy	Male (n = 450), n (%)	Female (n = 555), n (%)	Total (n = 1005), n (%)	p-value
Protein				
Insufficient	103 (22.9)	227 (40.9)	330 (32.8)	
Adequate	335 (74.4)	319 (57.5)	654 (65.1)	<0.05
Excess	12 (2.7)	9 (1.6)	21 (2.1)	
Lipid				
Insufficient	266 (59.1)	343 (61.8)	609 (60.6)	
Adequate	112 (24.9)	146 (26.3)	258 (25.7)	>0.05
Excess	72 (16.0)	66 (11.9)	138 (13.7)	
Carbohydrate				
Insufficient	274 (60.9)	429 (77.3)	703 (70.0)	
Adequate	114 (25.3)	92 (16.6)	206 (20.5)	<0.05
Excess	62 (13.8)	34 (6.1)	96 (9.6)	
Calcium adequacy				
Inadequate	441 (98.0)	528 (95.1)	969 (96.4)	<0.05
Adequate	9 (2.0)	27 (4.9)	36 (3.6)	
Iron adequacy				
Inadequate	41 (9.1)	192 (34.6)	233 (23.2)	<0.05
Adequate	409 (90.9)	363 (65.4)	772 (76.8)	
Zinc adequacy				
Inadequate	194 (43.1)	290 (52.3)	484 (48.2)	<0.05
Adequate	256 (56.9)	265 (47.7)	521 (51.8)	
Vitamin A adequacy				
Inadequate	346 (76.9)	445 (80.2)	791 (78.7)	>0.05
Adequate	104 (23.1)	110 (19.8)	214 (21.3)	
Folate adequacy				
Inadequate	366 (81.3)	495 (89.2)	861 (85.7)	<0.05
Adequate	84 (18.7)	60 (10.8)	144 (14.3)	
Vitamin C adequacy				
Inadequate	268 (59.6)	293 (52.8)	561 (55.8)	<0.05
Adequate	182 (40.4)	262 (47.2)	444 (44.2)	
